# The link between self-uncertainty and conspicuous consumption: Tolerance of uncertainty as a moderator

**DOI:** 10.3389/fpsyg.2022.1066938

**Published:** 2023-01-09

**Authors:** Xiaoming Wang, Hongjin Zhu, Qinying Zhao, Chaoqi Song, Xiuxin Wang

**Affiliations:** ^1^School of Psychology, Qufu Normal University, Qufu, China; ^2^Student Affairs Office, College Students Mental Health Education Center, Anyang Preschool Education College, Anyang, China

**Keywords:** self-uncertainty, conspicuous consumption, tolerance of uncertainty, uncertain context, compensatory consumption

## Abstract

This study, based on self-affirmation theory, aims to investigate the impact of self-uncertainty on individual consumption behavior. Self-uncertainty was categorized into moral, cognitive, and interpersonal self-uncertainty, and different types of self-uncertainty were manipulated through four experiments, including a moral dilemma, a recall paradigm, and a picture quiz task written by E-Prime software to examine the effects of different types of self-uncertainty on conspicuous consumption and their possible boundary conditions. Our results show that moral, cognitive, and interpersonal self-uncertainty contribute to a stronger tendency to engage in conspicuous consumption. Our results also suggest that tolerance of uncertainty moderates the effect of self-uncertainty on conspicuous consumption, meaning that subjects with a high tolerance of uncertainty are less inclined to engage in conspicuous consumption than those with a low tolerance of uncertainty, even if they have high self-uncertainty. This study may provide an explanation for conspicuous consumption behavior, further validating the theory of compensatory consumption. Additionally, the results from this study also provide a reference for understanding people’s decision-making behavior in an uncertain social context and can provide new guidance to control irrational consumption behavior.

## 1. Introduction

People sometimes seek out expensive goods, and the more expensive they are, the more they are loved by consumers, a phenomenon known as the “Veblen effect.” As economic development and material standards have increased, conspicuous consumption has received full attention in the fields of behavior, economics, and social psychology. Some researchers have argued that individuals engage in conspicuous consumption to highlight their social status, show their unique personalities, and protect their face and identity, which can negatively impact their social group and economic development (Leibenstein, 1950; Vigneron and Johnson, 1999; Trigg, 2001; O'Cass and McEwen, 2004; Deng and Dai, 2005). However, some studies suggest that conspicuous consumption is a way for individuals to consume symbolic goods or services to satisfy their needs in a particular context and that there may be positive effects (Rucker and Galinsky, 2008; Sivanathan and Pettit, 2010; Zheng and Peng, 2014; Li and Sun, 2016; Chen et al., 2018; Hammad and El-Bassiouny, 2018; Zheng et al., 2018; Oh, 2021).

Compensatory consumption theory states that an individual’s need for X can be solved by acquiring X or by acquiring Y. If it is solved by Y, this process is called “compensation” (Grunert, 1993). Previous research has found that conspicuous consumption can compensate for a missing need within an individual (Rucker and Galinsky, 2013) for example, individuals may engage in conspicuous consumption to compensate for threats to their self-esteem (Hammad and El-Bassiouny, 2018; Zheng et al., 2018; Oh, 2021). This may imply that conspicuous consumption is also a means of compensatory consumption (Mazzocco et al., 2012; Ma et al., 2019). It is thus hypothesized that individuals who have their egos threatened will also compensate for their feelings through conspicuous consumption behavior. Uncertain contexts can cause the individual’s ego to feel threatened by, for example, lacking a sense of belonging or a lack of control, creating a sense of self-uncertainty, which in turn forces the individual to seek to restore that certainty (Luhmann et al., 2011; Wagoner and Hogg, 2017). Therefore, what will an individual do after an uncertain context activates self-uncertainty? Will he or she choose conspicuous consumption?

Self-uncertainty is a subjective feeling in which the individual has doubts about his or her self-concept (Van den Bos, 2009). Self-affirmation theory states that individuals will consciously maintain their ego integrity (Sherman and Cohen, 2006). Therefore, when individuals have high levels of self-uncertainty, they will adopt compensatory strategies to compensate and repair themselves (McGregor et al., 2001; McGregor and Jordan, 2007; Wagoner and Hogg, 2017). Studies have found that when individuals are faced with uncertain contexts, they tend to self-repair through consumption behaviors (Cornil and Chandon, 2013; Chen et al., 2017; Liang et al., 2018; Liu et al., 2018; Ma et al., 2019), with conspicuous consumption being an effective tool (Mazzocco et al., 2012; Ma et al., 2019). This is because people are used to seeing objects as part of an extension of the self (Belk, 1988), and some products that have conspicuousness have a symbolic and emblematic value of manifesting and repairing the self (Bell and Dittmar, 2011; Koles et al., 2018). This means that individuals may engage in conspicuous consumption to assert themselves and compensate for a sense of lack after an uncertain context has triggered self-uncertainty (Morrison et al., 2012; Sun et al., 2020). However, other studies found that individuals with high levels of self-uncertainty avoid standing out and engage in group-aligned decision-making behaviors when the group position is ambiguous (Van Prooijen et al., 2004; Smith et al., 2007). More specifically, when self-uncertainty is activated, individuals are less inclined to engage in conspicuous consumption. Accordingly, this study hypothesizes that this difference is because people are exposed to different uncertain contexts in which there are different environmental factors that influence their behaviors (Yang et al., 2017). In other words, different uncertain contexts will trigger different types of self-uncertainty in individuals, which in turn leads to different compensatory behaviors.

Current research suggests that self-uncertainty is mainly derived from conflict situations (Yang et al., 2017), which mainly include cognitive conflict situations, moral conflict situations, and interpersonal conflict situations. Cognitive conflict situations refer to situations in which evidence or facts are incompatible with existing knowledge concepts (Lee et al., 2003), where cognition is not a cognitive resource in the broad sense (Botvinick et al., 2001), but rather a variety of knowledge content that individuals already have (Darnon, 2007). Moral conflict situations refer to situations in which there are possible negative consequences arising from moral dilemmas (Pavlish et al., 2014), whereas interpersonal conflict situations refer to situations in which interdependent parties become conflicted and negative and want to separate (Barki and Hartwick, 2004). Accordingly, this study classifies self-uncertainty into cognitive, moral, and interpersonal self-uncertainty. Cognitive self-uncertainty and moral self-uncertainty are more concerned with the psychological processes of the self. When individuals have high levels of cognitive and moral self-uncertainty, they will accentuate their ego and engage in conspicuous consumption behavior to compensate for their sense of control and belonging (Krohne, 1989; Morrison et al., 2012; Sun et al., 2020). Interpersonal self-uncertainty refers to an individual’s uncertainty about his or her relationship with others, his or her identity, and status in the group (Morrison et al., 2012). Individuals in group situations are more likely to act in a way that is consistent with the group to compensate for their sense of lack and therefore do not engage in conspicuous consumption (Van Prooijen et al., 2004; Smith et al., 2007). Accordingly, this study proposes the hypothesis that cognitive and moral self-uncertainty increases individuals’ conspicuous consumption in uncertain social contexts, while interpersonal self-uncertainty decreases individuals’ conspicuous consumption.

In a previous study, McGregor et al. (2010) found that tolerance of uncertainty influenced behavioral responses in individuals with high self-uncertainty. More precisely, individuals with low tolerance of uncertainty will be very averse to a variety of uncertain and ambiguous situations and will engage in more compensatory behaviors than individuals with high tolerance of uncertainty once their self-uncertainty is accepted and experienced (Butzer and Kuiper, 2006). Tolerance of uncertainty reflects individual differences in the tendency of cognitive, emotional, or behavioral responses to contexts of uncertainty (Zvolensky et al., 2010). Therefore, it is hypothesized that tolerance of uncertainty moderates the effect of self-uncertainty on conspicuous consumption. That is, individuals with low tolerance of uncertainty are more likely to consume conspicuously than individuals with high tolerance of uncertainty.

**Figure 1:**
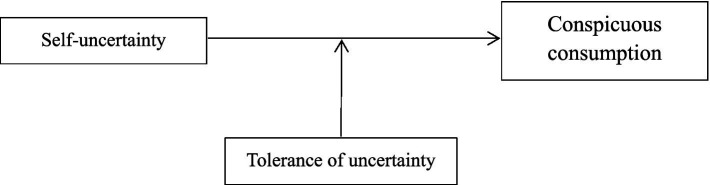
A theoretical model of the moderating effect of tolerance of uncertainty.

In summary, based on compensatory consumption theory and self-affirmation theory, this study proposes to examine the influence of moral, cognitive, and interpersonal self-uncertainty on conspicuous consumption through four experiments while introducing tolerance of uncertainty as a moderating variable to further investigate the influence of self-uncertainty on conspicuous consumption (see Figure 1). This study explores self-uncertainty from three conflict contexts, aiming to investigate the impact of uncertainty on individuals’ behavioral decisions and to verify the compensatory role of conspicuous consumption. It is also expected to provide new ideas on individual behavior and decision-making in uncertain social contexts and to draw attention to the real needs of the inner self.

## 2. Experiment 1: The effect of moral self-uncertainty on conspicuous consumption

### 2.1. Pilot study

Self-uncertainty is derived from uncertain social contexts so experiment 1 aimed to initiate moral self-uncertainty through a moral dilemma (Yang et al., 2017). The initiation of moral self-uncertainty in this experiment was mainly reflected in the choice of giving up one’s seat to whichever person the subject chose to give up their seat to; the absence of the other person caused a conflict on the subject’s moral level. Disabled people, pregnant women, and the elderly are considered to be representatives of vulnerable passengers, while the vulnerability of disabled people is more likely to be perceived (Stjernborg, 2019). So, in the pilot study, different groups of subjects made a choice to give up their seats among different groups of objects in the same situation to further clarify which group of objects was more capable of triggering the subjects’ uncertainty (Gawronski et al., 2017).

In the pilot study, a total of 62 subjects were recruited and randomly assigned to Group A and Group B. In Group A, subjects were asked to choose to give up their seats to a pregnant woman or a disabled person on a bus. The subjects in Group A were required to choose to give up their seats to a pregnant woman or a disabled person on a bus, while the subjects in Group B were required to choose to give up their seats to an elderly person or a disabled person, after which they completed the self-uncertainty scale (Yang et al., 2019). An independent-samples *t*-test was used to analyze the data. The results revealed that subjects in Group A (*M* = 4.26, *SD* = 1.65) had significantly higher moral uncertainty than those in Group B (*M* = 3.16, *SD* = 1.64), *t*_(184)_ = 2.63, *p* < 0.05, Cohen’s *d* = 0.67. Accordingly, pregnant women and disabled people will be the subjects who give up their seats in experiment 1.

### 2.2. Participants

The sample size required for the experiment, as calculated by G^*^power, was 128 when the level of significance α = 0.05 and the 80% statistical power level was reached. A total of 187 university students (40 men, 147 women; *M_age_* = 19.16, *SD_age_* = 1.23) were recruited for experiment 1 and were randomly assigned to the experimental (*n* = 94) and control groups (*n* = 92). All enrolled participants provided informed consent prior to the start of the study and were able to understand the experimental material correctly.

### 2.3. Experimental design and materials

The experiment used a one-factor (moral self-determination: experimental group vs. control group) between-subjects design. Participants in the experimental group were required to choose to give up their seats for a pregnant woman or a disabled person, whereas participants in the control group were not required to face such a moral dilemma and were simply required to give up their seats to a single subject.

The manipulation check of self-uncertainty was carried out by presenting subjects with 15 words describing emotions (6 positive words: secure, excellent, happy, smart, successful, lovable, meaningful; 6 negative words: depressed, empty, lonely, ashamed, stupid, confused, anxious; and 1 word related to the independent variable: uncertainty) and asking them to judge the intensity of the word corresponding to the emotion (Watson et al., 1988; Yang et al., 2019). The questionnaire used a 7-point Likert scale (1 = “extremely weak,” 7 = “extremely strong”).

The measure of the conspicuous consumption tendency was adapted from the conspicuous consumption scale. (It is important to know what friends think of different brands and categories before buying a product; it is important to know who buys the brand and category; it is important to know what others think of the person using the brand or category; it is important to be able to make a good impression; and I used to buy expensive brands just because I knew people would notice it; I love the prestige that comes with buying a brand). developed by Marcoux et al. (1997), which consists of six questions, each using a 5-point Likert scale (1 = “strongly disagree,” 5 = “strongly agree”). Cronbach’s α = 0.628 for this scale in this study.

### 2.4. Experimental procedures

The participants entered the laboratory, where they were informed of the purpose of the experiment and the principle of confidentiality and were randomly assigned to either the experimental or control group. Participants then read a section of the bus seat concessions scenario, where participants in the experimental group were required to make a choice and participants in the control group were not required to make a choice. The participants were then asked to complete a manipulation check task and finally fill in the conspicuous consumption scale and some basic information from the demographic survey.

### 2.5. Results

#### 2.5.1. Manipulation check

An independent-samples *t*-test of the scores of participants in both groups on the self-uncertainty manipulation check question indicated that moral uncertainty was significantly higher in the experimental group (*M* = 4.37, *SD* = 1.67) than in the control group (*M* = 2.28, *SD* = 1.60), *t*_(184)_ = 8.73, *p* < 0.001, Cohen’s *d* = 1.28. This suggests that the manipulation of moral uncertainty was effective.

#### 2.5.2. The effect of moral self-uncertainty on conspicuous consumption

An independent-samples *t*-test of the scores on the conspicuous consumption tendency showed that subjects in the experimental group (*M* = 19.02, *SD* = 3.13) had a significantly higher propensity to consume conspicuously than those in the control group (*M* = 17.87, *SD* = 3.18), *t*_(184)_ = 2.50, *p* = 0.014, Cohen’s *d* = 0.36. This indicates that in a state of moral self-uncertainty, individuals’ conspicuous consumption increases. This also provides supplemental evidence that individuals’ conspicuous consumption is an attempt to compensate for their uncertainty and restore a sense of certainty on the moral level.

### 2.6. Discussion

Experiment 1 verified that moral self-uncertainty has an impact on conspicuous consumption because individuals in a state of uncertainty feel a lack of self-control and a lack of control over their own morality and use conspicuous consumption to compensate for this lack of need (Langer, 1975; Sherman and Cohen, 2006; Wagoner and Hogg, 2017). In addition, research has found that individuals feel uncertain not only because of their own behavior but also because of cognitive conflicts and interpersonal relationships (Morrison et al., 2012). Therefore, Experiments 2–3 will explore the effects of different types of self-uncertainty on conspicuous consumption behavior, both in terms of cognitive and interpersonal relationships.

## 3. Experiment 2: The effect of cognitive self-uncertainty on conspicuous consumption

### 3.1. Participants

The sample size required for the experiment, as calculated by G^*^power, was 128 when the level of significance α = 0.05 and the 80% statistical power level was reached. A total of 185 university students (62 men, 123 women; *M_age_* = 19.84, *SD_age_* = 1.29) were recruited for experiment 2 and were randomly assigned to the experimental (*n* = 92) and control groups (*n* = 93). All enrolled participants provided informed consent prior to the start of the study and were able to understand the experimental material correctly. None of the participants had participated in similar experiments.

### 3.2. Experimental design and materials

**Figure 2:**
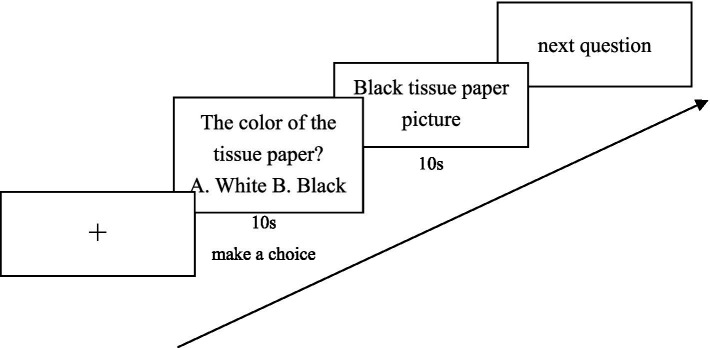
Flowchart of experiment 2 section.

Experiment 2 used a one-factor (cognitive self-uncertainty: experimental group vs. control group) between-subjects experimental design in which cognitive self-uncertainty was initiated by a picture quiz question written by E-Prime software. This was done by first presenting a general knowledge question on the screen, after which it would move to the self-uncertainty manipulation. In the manipulation, there were pictures presented on the screen describing things (the experimental group saw things that were contrary to common knowledge, and the control group saw things that were consistent with common knowledge) and then participants were asked to look closely at the pictures for 10 s to respond, with a total of 11 questions (see Figure 2; Taylor and Noseworthy, 2020).

Contextual tests were used to test the conspicuous consumption tendency (Goor et al., 2020). Participants answered 2 questions after reading a consumption scenario in which they purchased a suit to wear to a party: “What price point suit do you think will get you more attention?” and “At what price point do you think a suit would enhance your image in the eyes of others? (1 = “Prefer a $500 suit,” 7 = “Prefer a 1,500 suit”).

### 3.3. Experimental procedures

Participants entered the laboratory and completed the experiment on a computer. First, an instructional message was presented on the screen informing participants of the purpose of the experiment and the principle of confidentiality, and they were randomly assigned to either the experimental group or the control group. Participants then completed 11 general knowledge multiple choice questions, with participants in the experimental group selecting a picture on the screen that did not match their general knowledge and participants in the control group seeing a picture that matched their general knowledge. Participants then had to complete a manipulation check task, and those who did not pass the manipulation check were excluded. Finally, participants were then asked to complete the contexts of conspicuous consumption test as well as some basic information from the demographic survey.

### 3.4. Results

#### 3.4.1. Manipulation check

An independent-samples *t*-test of the scores of participants in both groups on the manipulation check question on cognitive self-uncertainty indicated that cognitive uncertainty was significantly higher in the experimental group (*M* = 3.15, *SD* = 2.01) than in the control group (*M* = 2.18, *SD* = 1.50), *t*_(183)_ = 3.73, *p* < 0.001, Cohen’s *d* = 0.55. This suggests that the manipulation of cognitive self-uncertainty was effective.

#### 3.4.2. The effect of cognitive self-uncertainty on conspicuous consumption

An independent-samples *t*-test of the scores on the conspicuous consumption tendency showed that the propensity to consume ostentatiously was significantly higher in the experimental group (*M* = 8.98, *SD* = 2.84) than in the control group (*M* = 7.94, *SD* = 3.32), *t*_(183)_ = 2.30, *p* < 0.05, Cohen’s *d* = 0.34. This suggests that individuals under perceived self-uncertainty engage in more conspicuous consumption.

### 3.5. Discussion

The results of Experiment 2 validated the effect of cognitive self-uncertainty on conspicuous consumption behavior, where individuals adopted more conspicuous consumption behavior when cognitive self-uncertainty was activated. The results of the experiment also reaffirm the self-affirmation theory and the compensatory consumption theory. Both theories suggest that individuals will respond in other ways if they cannot directly eliminate threats to the ego or satisfy missing needs (Sherman and Cohen, 2006; Rucker and Galinsky, 2013). The increase in the propensity to conspicuous consumption suggests that subjects respond to perceived ego uncertainty by conspicuous consumption, implying that conspicuous consumption is an effective means of compensation in uncertain contexts and can help individuals cope with the effects of uncertain contexts (Mazzocco et al., 2012; Cornil and Chandon, 2013; Chen et al., 2017; Liang et al., 2018; Liu et al., 2018; Ma et al., 2019). After seeing pictures that contradict general knowledge, participants had doubts about their own cognition and consequently felt that their surroundings and the world were difficult to control, but they were unable to take direct measures to restore their sense of control and their ego were also threatened; therefore, participants need to compensate for their lack of self through conspicuous consumption (Mazzocco et al., 2012; Wagoner and Hogg, 2017; Ma et al., 2019).

## 4. Experiment 3: The effect of interpersonal self-uncertainty on conspicuous consumption

### 4.1. Participants

The sample size required for the experiment, as calculated by G^*^power, was 128 when the level of significance α = 0.05 and the 80% statistical power level was reached. A total of 188 university students (52 men, 136 women; *M_age_* = 19.57, *SD_age_* = 1.30) were recruited for experiment 3 and were randomly assigned to the experimental (*n* = 94) and control groups (*n* = 94). All enrolled participants provided informed consent prior to the start of the study and were able to understand the experimental material correctly.

### 4.2. Experimental design and materials

Experiment 3 used a one-factor (interpersonal self-uncertainty: experimental group vs. control group) between-subjects design. This experiment was designed to initiate interpersonal self-uncertainty through a recall paradigm in which subjects in the experimental group were asked to recall an experience in which they were rejected or betrayed and to write down the experience in detail, while subjects in the control group were asked to recall an experience in which they felt warm and harmonious and to write down the experience (Hohman and Hogg, 2015; Sun et al., 2020). The independent variable manipulation checks were the same as in experiment 1.

The measure of conspicuous consumption was a combination of a scale and a contextual test. The scale test was identical to experiment 1, and the questions on the situational test were identical to experiment 2 but were scored using a dichotomous approach, i.e., The participants were given a direct choice between two price sets (1 = Set A, 2 = Set B).

### 4.3. Experimental procedures

Participants who entered the laboratory alone were informed of the purpose of the experiment and the principle of confidentiality and were randomly assigned to either the experimental or control group. Participants in the experimental group were then asked to recall an experience of rejection or betrayal, and participants in the control group were asked to recall an experience in which they felt warm and harmonious. Afterward, participants were required to complete an independent variable manipulation check. Finally, participants were asked to complete the conspicuous consumption scale and the conspicuous consumption context test, as well as some basic information from the demographic survey.

### 4.4. Results

#### 4.4.1. Manipulation check

An independent-samples *t-*test of the scores on the manipulation check question on interpersonal self-uncertainty for both groups of subjects indicated that the experimental group had significantly higher interpersonal self-uncertainty (*M* = 3.60, *SD* = 1.76) than the control group (*M* = 2.67, *SD* = 1.51), *t*_(186)_ = 3.90, *p* < 0.001, Cohen’s *d* = 0.57. This suggests that the manipulation of interpersonal self-uncertainty was effective.

#### 4.4.2. The effect of interpersonal self-uncertainty on conspicuous consumption

A Chi-square test was conducted on the contextual test score for the conspicuous consumption tendency. The results found that the premises of the Chi-square test of independence were met in the contextual test with a sample size of 186 > 40 and no cells with an expectation value <5, *χ^2^*(1, *N* = 186) = 6.98, *p* < 0.05. This indicated that there was a significant difference between the experimental and control groups.

An independent-samples *t-*test of the test scores for conspicuous consumption showed that subjects in the experimental group (*M* = 19.13, *SD* = 3.02) had a higher propensity for conspicuous consumption than those in the control group (*M* = 17.53, *SD* = 3.40), *t*_(186)_ = 3.40, *p* < 0.001, Cohen’s *d* = 0.50. In other words, individuals with higher interpersonal self-uncertainty (experimental group) had a stronger propensity for conspicuous consumption than those with lower interpersonal self-uncertainty (control group).

### 4.5. Discussion

The hypothesis of this study suggested that interpersonal self-uncertainty would reduce individuals’ conspicuous consumption, whereas the results of experiment 3 were inconsistent with the hypothesis. This may be because individuals can compensate themselves in a variety of ways under uncertainty, and conspicuous consumption can also be used to respond to different situations and different sources of threat (Sun et al., 2021). When interpersonal self-uncertainty is activated, individuals feel that their social identity is threatened, and to prove that they have good qualities and thus gain a sense of control and belonging, they will engage in more conspicuous consumption (Li et al., 2017). The results of all three experiments show that individuals in a state of ego uncertainty will engage in more conspicuous consumption and that different types of self-uncertainty can lead to a greater tendency toward conspicuous consumption. At the same time, the results of the experiment also confirm the positive effect of conspicuous consumption, as people engage in conspicuous consumption to compensate for their sense of lack.

To examine the factors influencing individual behavior in uncertain contexts and to help individuals clarify the nature behind their behavior, experiment 4 will further explore the possible boundary conditions between self-uncertainty and conspicuous consumption. In this study, moral and cognitive self-uncertainty are only related to individuals’ knowledge and values, whereas interpersonal self-uncertainty is not only closely related to the self but also involves social dimensions, so this study considers interpersonal self-uncertainty to be representative. Therefore, experiment 4 builds on the findings of experiment 3 to further investigate how tolerance of uncertainty plays a moderating role in the process of self-uncertainty influencing conspicuous consumption.

## 5. Experiment 4: The effect of self-uncertainty on conspicuous consumption—The role of tolerance of uncertainty

### 5.1. Participants

The sample size required for the experiment, as calculated by G^*^power, was 179 when the level of significance α = 0.05 and the 80% statistical power level was reached. A total of 215 university students (59 men, 156 women; *M_age_* = 19.58, *SD_age_* = 1.38) were recruited for experiment 4 and were randomly assigned to the experimental (*n* = 103) and control groups (*n* = 111). All enrolled participants provided informed consent prior to the start of the study and were able to understand the experimental material correctly.

### 5.2. Experimental design and materials

This experiment used a one-factor (interpersonal self-uncertainty: experimental group vs. control group) between-subjects design. Tolerance of uncertainty was measured using the Chinese version of The Intolerance of Uncertainty Scale-12 (IUS-12; Zhang et al., 2017), which is scored on a 5-point Likert scale (1 = “not at all characteristic of me,” 5 = “very characteristic of me”), with all questions reverse scored, and the scale had a Cronbach’s α = 0.800 in this study.

### 5.3. Experimental procedures

Participants who entered the laboratory alone were informed of the purpose of the experiment and the principle of confidentiality and were randomly assigned to either the experimental or control group. Participants in the experimental group were then asked to recall an experience of rejection or betrayal, and participants in the control group were asked to recall an experience in which they felt warm and harmonious. Afterward, participants were required to complete an independent variable manipulation check and then received a measure of tolerance of uncertainty. Finally, participants were asked to complete the conspicuous consumption scale, as well as some basic information from the demographic survey.

### 5.4. Results

#### 5.4.1. Manipulation check

An independent-samples *t*-test of the scores on the manipulative check question on interpersonal self-uncertainty for both groups of subjects indicated that the experimental group had significantly higher interpersonal uncertainty (*M* = 3.56, *SD* = 1.74) than the control group (*M* = 2.96, *SD* = 1.65), *t*_(212)_ = 2.60, *p* < 0.05, Cohen’s *d* = 0.35. This suggests that the manipulation of interpersonal self-uncertainty was effective.

#### 5.4.2. The effect of interpersonal self-uncertainty on conspicuous consumption

An independent-samples *t*-test of the scores on the conspicuous consumption tendency showed that the propensity to consume in an ostentatious manner was higher in the experimental group (*M* = 18.84, *SD* = 3.17) than in the control group (*M* = 17.89, *SD* = 3.40), *t*_(212)_ = 2.11, *p* < 0.05, Cohen’s *d* = 0.29 (see Table 1).

**Table 1:** The effect of interpersonal self-uncertainty on conspicuous consumption in experiment 4.

Variables (V)	Experimental group (*M* ± *SD*)	Control group (*M* ± *SD*)	*t*	*p*	Cohen’s *d*
Self-uncertainty	3.56 ± 1.74	2.96 ± 1.65	2.60	0.010	0.35
Conspicuous consumption	18.84 ± 3.17	16.27 ± 3.40	2.11	0.035	0.29

#### 5.4.3. The moderating role of tolerance of uncertainty

**Figure 3:**
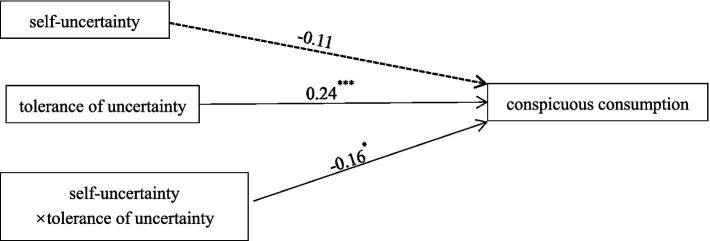
Statistical model of the moderating effect of tolerance of uncertainty. The solid line in the graph indicates that *p* < 0.05; the dashed line indicates that ^*^p < 0.05; ^**^p < 0.01; ^***^p < 0.001.

Interpersonal self-uncertainty (experimental group = 1, control group = 0) was used as the independent variable, tolerance of uncertainty as the moderating variable, and conspicuous consumption behavior as the dependent variable. Process v 3.4 (Model 8) was used to test the moderating effect of tolerance of uncertainty on self-uncertainty and conspicuous consumption (Hayes, 2013; see Figure 3). Standardized data were used in this study. It was found that the interaction between interpersonal self-uncertainty and tolerance of uncertainty significantly predicted conspicuous consumption behavior (β = −0.16, *SE* = 0.07, *t* = −2.38, *p* < 0.05, *95%* CI [−0.29, −0.03]).

**Figure 4:**
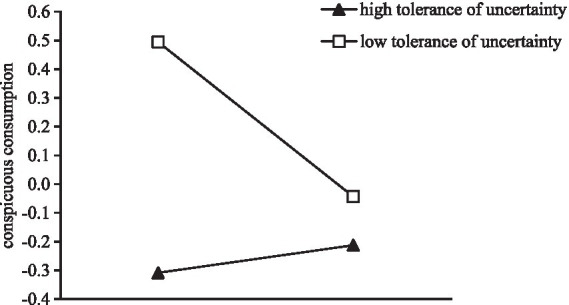
A simple slope of tolerance of uncertainty.

Further simple slope analysis showed that there was no significant difference between the experimental and control groups’ conspicuous consumption behavior in the high tolerance of uncertainty condition [β = 0.05, *SE* = 0.09, *t* = 0.51, *p* > 0.05, *95%* CI (−0.14, 0.23)], while in the low tolerance of uncertainty condition, the experimental group’s propensity to conspicuous consumption was significantly higher than the control group’s propensity to conspicuous consumption (β = −0.27, *SE* = 0.09, *t* = −2.88, *p* < 0.001, *95%* CI [−0.45, −0.08]; see Figure 4).

## 6. General discussion

Individual behavioral decisions in uncertain contexts have received extensive attention in behavior, economics, and social psychology. It has been found that uncertain contexts pose a threat to individuals, and to compensate for this threat, individuals will adopt some behaviors to compensate for it (Choi et al., 2019; Dufner et al., 2019), and conspicuous consumption can be a good way to compensate for the sense of lack triggered by self-uncertainty (Mazzocco et al., 2012; Ma et al., 2019). Therefore, this study provides insight into the effects of self-uncertainty on conspicuous consumption by using different methods to manipulate different types of self-uncertainty and examines the moderating role of tolerance of uncertainty.

First, experiments 1–3 primarily explored the effects of moral, cognitive, and interpersonal self-uncertainty on conspicuous consumption, with results indicating that moral, cognitive, and interpersonal self-uncertainty all motivate individuals to engage in conspicuous consumption. The results of experiments 1–2 were consistent with the expectations, finding that moral and cognitive self-uncertainty lead individuals to engage in conspicuous consumption. This is possibly because when individuals develop feelings of uncertainty, they actively take means to compensate for them, and conspicuous consumption is an effective means for individuals to restore their sense of certainty (Mazzocco et al., 2012; Ma et al., 2019). However, the results of experiment 3 were contrary to the expectations, and it was found that interpersonal self-uncertainty triggered conspicuous consumption. This may be because, in experiment 3, participants were confronted with a different uncertainty context than in previous studies. Whereas the situation in the previous study was merely one in which participants felt inconsistent with the group, and this inconsistency which may not have threatened their status and did not constitute interpersonal conflict may have led individuals to seek group approval (Hogg, 2007), the situation in experiment 3 accurately manipulated participants’ interpersonal self-uncertainty. The participants may feel less valued in the group and that their status was threatened by recalling their experiences of exclusion or neglect (Shin et al., 2020; Yang et al., 2020), and instead of choosing to conform to the group (Smith et al., 2007), they may choose to consume conspicuously to manifest their self-worth and increase their sense of self-worth (Morrison et al., 2012; Sun et al., 2020). Self-affirmation theory also suggests that individuals compensate for a lack of sense of worth by maintaining a good self-image (Sherman and Cohen, 2006; Teng, 2019) and that the visibility and uniqueness of conspicuous goods can be well suited to the individual’s purpose of maintaining a good image (Bell and Dittmar, 2011; Koles et al., 2018). At the same time, after interpersonal self-uncertainty is activated, individuals may also feel that their social identity is threatened and lose their sense of control over their surroundings and sense of belonging in the group, but individuals cannot immediately regain their sense of control and belonging and must take other means to compensate themselves, and conspicuous consumption can help individuals compensate for the lack of feeling (Li et al., 2017). Thus, individuals who lack cognitive self-uncertainty, moral self-uncertainty, or interpersonal self-uncertainty will compensate for different uncertain social contexts through conspicuous consumption (Mazzocco et al., 2012; Ma et al., 2019; Sun et al., 2021).

Furthermore, experiment 4 investigated the moderating role of tolerance of uncertainty in the effect of self-uncertainty on conspicuous consumption. The results showed that individuals with a high tolerance of uncertainty were significantly less inclined to conspicuous consumption than the control group, even when they were in a state of high self-uncertainty (experimental group). Perhaps, this is because individuals with a high tolerance of uncertainty are better able to accept uncertainty in the face of uncertainty contexts, and they are able to suppress the negative emotions caused by self-uncertainty to a certain extent, thus reducing compensatory behavior (Bredemeier and Berenbaum, 2008; Di Trani et al., 2021). Individuals with a low tolerance of uncertainty are more likely to experience worry and anxiety in uncertain contexts (McEvoy and Mahoney, 2012; Boelen et al., 2014) and are more sensitive to uncertainty and intolerant of it (Ladouceur et al., 1997, 2000). Therefore, some means of alleviating self-uncertainty are necessary. On this basis, there is some reason to hypothesize that tolerance of uncertainty is a key factor influencing individuals’ behavioral decisions in uncertain contexts.

The findings of this study are also consistent with the uncertainty theory proposed by previous studies. Uncertainty-identity theory views self-uncertainty as a feeling of doubt about the self, the world view and the environment in which one lives (Van den Bos, 2001), causing disgust and discomfort (Hogg, 2007) and thus a loss of control and certainty over one’s environment (Van den Bos, 2007). When individuals are in a state of self-uncertainty, feelings of discomfort and disgust drive individuals’ motivation to reduce self-uncertainty (Van den Bos, 2001; Van den Bos, 2009). In turn, to protect the self from uncertainty, individuals turn to material objects for alleviation, resulting in a state of self-stability and coherence (Chang and Arkin, 2002; Rucker and Galinsky, 2008; Gao et al., 2009). As a result, subjects showed more propensity to conspicuous consumption in order to remove uncertainty. Both the Uncertainty Management Model and the Uncertain Response Convergence Motivation Theory emphasize that in order to alleviate self-uncertainty, people will generate and pursue needs in other areas in order to reduce uncertainty (Van den Bos and Lind, 2002; McGregor et al., 2010). Conspicuous consumption is a way of showing off one’s uniqueness and distinguishing oneself, so people use it to counteract the uncertainty of uncertain situations (Bell and Dittmar, 2011; Koles et al., 2018). The findings also suggest that conspicuous consumption may have a compensatory effect and that it also has some positive effects on people’s daily lives, especially in uncertain contexts. Despite its negative perception, conspicuous consumption can go some way to curbing the worry and anxiety caused by self-uncertainty, helping people to tolerate the aversion caused by uncertain contexts and adapt better to this age of information explosion (Bredemeier and Berenbaum, 2008; Luhmann et al., 2011; Carleton, 2012). Furthermore, the results of this study also suggest that compensatory behavior may be the main means by which people cope with uncertainty, with conspicuous consumption being only one way of doing so (Hammad and El-Bassiouny, 2018; Zheng et al., 2018; Oh, 2021). In uncertain contexts, compensating for one’s inner needs may be a common behavioral norm (McGregor et al., 2001; McGregor and Jordan, 2007; Smith et al., 2007; Wagoner and Hogg, 2017). Future research could explore the psychological mechanisms of people’s compensatory behavior in uncertain contexts.

This study innovatively proposes three types of self-uncertainty, focuses on the impact of different types of situations on individuals, and explores in depth the behavioral tendencies of individuals in different uncertain situations. Future research can pay more attention to the differences in individuals’ behavioral decisions under different uncertain social situations. In addition, this study verifies the positive effect of conspicuous consumption, which provides some reference for understanding individual behavior in uncertain situations and contributes to people’s view of their true inner needs. Of course, this study also has certain limitations. First, in exploring the moderating role, this study used only interpersonal self-uncertainty and did not explore in more detail the role played by moral uncertainty and cognitive uncertainty in this pathway. Subsequent research could explore whether there are significant differences between these two aspects of self-uncertainty in the regulation mechanism. Second, this study only explored the moderating mechanisms by which self-uncertainty influences conspicuous consumption, without engaging in a discussion of mediating effects. For example, among the concepts that are highly correlated with self-uncertainty, self-esteem is a variable that has received keen attention, and future research could explore it more deeply in relation to state self-esteem.

## 7. Conclusion

The study explored the effects of self-uncertainty and tolerance of uncertainty on conspicuous consumption through a moral dilemma, a recall paradigm, and a picture quiz task written by E-Prime software. It was found that (1) both moral self-uncertainty and cognitive self-uncertainty made individuals more inclined to conspicuous consumption; (2) individuals’ conspicuous consumption increased when interpersonal self-uncertainty was activated; and (3) tolerance of uncertainty moderated the effect of self-uncertainty on conspicuous consumption, as individuals with low tolerance of uncertainty showed a greater tendency for conspicuous consumption when their self-uncertainty was activated compared to those with higher tolerance of uncertainty.

## Data availability statement

The raw data supporting the conclusions of this article will be made available by the authors, without undue reservation.

## Ethics statement

The studies involving human participants were reviewed and approved by Qufu Normal University. The patients/participants provided their written informed consent to participate in this study.

## Author contributions

XMW for variables, methods, and article writing. CQS for providing materials of the four experiments and assistance with data collection. HJZ and QYZ for revision assistance. XXW for helpful suggestions about the experimental design. All authors contributed to the article and approved the submitted version.

## Conflict of interest

The authors declare that the research was conducted in the absence of any commercial or financial relationships that could be construed as a potential conflict of interest.

## Publisher’s note

All claims expressed in this article are solely those of the authors and do not necessarily represent those of their affiliated organizations, or those of the publisher, the editors and the reviewers. Any product that may be evaluated in this article, or claim that may be made by its manufacturer, is not guaranteed or endorsed by the publisher.
